# *g* versus *c*: comparing individual and collective intelligence across two meta-analyses

**DOI:** 10.1186/s41235-021-00285-2

**Published:** 2021-04-03

**Authors:** Luke I. Rowe, John Hattie, Robert Hester

**Affiliations:** 1 National School of Education, Australian Catholic University, East Melbourne, VIC Australia; 2 Science of Learning Research Centre, The University of Melbourne, Parkville, VIC Australia; 3 School of Psychological Sciences, The University of Melbourne, Parkville, VIC Australia

**Keywords:** Collective intelligence, *C-*factor, *G-*factor, IQ, Group performance

## Abstract

Collective intelligence (CI) is said to manifest in a group’s domain general mental ability. It can be measured across a battery of group IQ tests and statistically reduced to a latent factor called the “*c-*factor.” Advocates have found the *c-*factor predicts group performance better than individual IQ. We test this claim by meta-analyzing correlations between the *c-*factor and nine group performance criterion tasks generated by eight independent samples (*N* = 857 groups). Results indicated a moderate correlation, *r*, of .26 (95% CI .10, .40). All but four studies comprising five independent samples (*N* = 366 groups) failed to control for the intelligence of individual members using individual IQ scores or their statistically reduced equivalent (i.e., the *g-*factor). A meta-analysis of this subset of studies found the average IQ of the groups’ members had little to no correlation with group performance (*r* = .06, 95% CI −.08, .20). Around 80% of studies did not have enough statistical power to reliably detect correlations between the primary predictor variables and the criterion tasks. Though some of our findings are consistent with claims that a general factor of group performance may exist and relate positively to group performance, limitations suggest alternative explanations cannot be dismissed. We caution against prematurely embracing notions of the *c-*factor unless it can be independently and robustly replicated and demonstrated to be incrementally valid beyond the *g-*factor in group performance contexts.

## Significance statement

In 2010 Woolley, Chabris, Pentland, Hashmi, and Malone provided evidence for the existence of a general factor of collective intelligence in groups, the *c-*factor. The *c-*factor was purportedly analogous to but empirically distinct from the *g-*factor—a well-established and validated general factor of intelligence in individuals. Authors in support of the *c-*factor have rightly claimed that validating such a factor could have far-reaching theoretical and practical implications. Selecting and screening for high-IQ *individuals*, for example, is a common practice that could potentially be usurped in socially intensive contexts by instead selecting, screening for, and promoting high-IQ *groups*. The meta-analyses and broader quantitative syntheses provide the backdrop for a critical review of the validity of the *c-*factor and how it compares to the *g*-factor in group performance settings. While the present results suggest the *c-*factor may indeed correlate moderately with group performance in specific research contexts better than the IQ of the groups’ individual members, available studies are relatively sparse, statistically underpowered, and methodologically problematic. Taking these issues into consideration, we advise potential adoptees of practices that aim to develop higher performing groups, such as organizations, medical institutions, and schools, to refrain from embracing the *c-*factor unless further evidence accumulates in its favor. Instead we recommend researchers and practitioners continue to measure and account for intelligence in groups using individual IQ tests validated around psychometric *g*.

## *g* versus *c*: two meta-analyses

Intelligence is a term often used to describe an individual’s capacity to make sense of complexity, deal with indeterminacy and novelty, reason from problems to solutions, comprehend ideas, learn and adapt to an ever-changing environment, and to do all of this with optimal efficiency (Euler 2018; Gottfredson 1998; Neisser et al. 1996). The scientific study of intelligence often compares individual differences in these capacities and owes its origins story to the theory of general intelligence; formally posited by Charles Spearman in 1904 and further developed in 1927 in an attempt to explain positive correlations among test results across a variety of academic domains (e.g., mathematics, languages) (Spearman 1904,1927). The correlations enabled Spearman to perform one of the earliest forms of factor analysis and extract a single, *general* factor of intelligence labeled Spearman’s *g* or simply the “*g-*factor.” Despite its chequered history (Carson 2015; Fletcher and Hattie 2011), the *g-*factor has provided the underlying theory guiding many modern intelligence tests (e.g., McGrew et al. 2014; Raven 1998; Tulsky et al. 1997; Wonderlic 1992) and proven useful explaining and predicting differences among individuals across academic (Rohde and Thompson 2007), occupational (Schmidt et al. 2016), and health-related (Deary and Batty 2011) outcomes, and have purportedly saved billions of dollars for organizations willing to utilize them for recruitment and selection purposes (Schmidt and Hunter 1981).

A notable area to which the notion of general intelligence has been applied over recent decades is the field of group performance. There have been at least three meta-analyses since the year 2000 examining the effect of general intelligence on group performance (Bell 2007; Devine and Philips 2001; Stewart 2006). Across these meta-analyses, the average sample-weighted correlation between the average IQ of the groups’ individual members and group performance is .28 (95% CI .25, .30).

These findings have not deterred attempts to develop novel ways of measuring intelligence in groups. One example comes from a highly cited article by Woolley et al. (2010), where they claimed to have found a superior approach to explaining and predicting group performance by measuring the groups’ collective intelligence. This approach, which we refer to as the group IQ paradigm, involves administering to groups a variety of mental tasks in an IQ test-like format. Factor analysis is then applied to the test results, and from the intercorrelations among them, a general factor is statistically extracted. Because the factor contains variance that is common among the variety of tasks and is a product of inputs from multiple members, it can be thought of as a *general collective intelligence factor* analogous to Spearman’s *g-*factor. This factor, labeled hereafter as the *c-*f*actor*, represents the *group’s domain-general mental ability* and is distinguished from other group-related phenomena when “the ability of a group to perform one task is correlated with that group’s ability to perform a wide range of other tasks” (Woolley et al. 2010, p. 687).

Woolley et al. (2010) outlined three additional statistical and theoretical criteria, paralleling those used to establish the *g-*factor (Chabris 2007), crucial to an empirical account of the *c-*factor. Firstly, from the inter-correlations among an omnibus of mental tests administered to groups (under the group IQ paradigm), an exploratory factor analysis must yield a single factor explaining 30–50% of variance in performance. Secondly, this single factor will explain at least double the variance compared to the next largest factor. Thirdly and finally, this factor cannot be otherwise accounted for (partially or fully) by other plausible alternatives such as the average intelligence of a group’s individual members (i.e., the *g-*factor).

While no clear boundaries exist in relation to what constitutes a valid unit of analysis under this paradigm, studies asserting the existence of the *c-*factor typically operationalize it in groups of two to six people working jointly on a battery of group IQ tests lasting no longer than one to two hours. These have typically comprised five to ten tasks such as group matrix reasoning, a planned shopping and expenditure task, group brainstorming, and group unscrambling of words (see Credé and Howardson 2017a, b, p. 1484 for an excellent summary). Measuring group ability via the *c-*factor typically involves members synchronously and freely discussing test items in online and face-to-face settings, with spoken or written communication, and with members that are familiar or unfamiliar to them. This approach to measuring group ability differs considerably from “crowd IQ,” “wisdom of crowds,” or Delphi-type methods which often involve establishing and following guidelines on how members interact, divide labor, communicate and elicit ideas, and synthesize responses (e.g., Hemming et al. 2018; Kosinski et al. 2012; Surowiecki 2005).

Since it was first hypothesized in 2010, the *c-*factor has been conceptually replicated by the original authors across different cultures (Engel et al. 2015a, b) and contexts (Engel et al. 2014a, b) and has been reported as sharing links to a variety of real-world outcomes including business performance (Mayo and Woolley 2017), team learning and academic achievement (Engel et al. 2015a, b; Woolley and Aggarwal 2017), scientific endeavors (Bear and Woolley 2011; Woolley and Fuchs 2011), competitive team video-game performance (Kim et al. 2017a, b), and high-stakes group decision-making (Radcliffe et al. 2019; Rogers et al. 2019).

## Significance and research questions

The emerging body of research in support of a *c-*factor is particularly surprising because, until recently, the majority view suggested a group’s ability to perform a given task is situationally specific (Cohen and Bailey 1997; Devine 2002). According to the situationally specific perspective, a group that thrives in one undertaking, such as producing intelligence reports, may not necessarily thrive doing other undertakings, such as developing a strategy document or solving a complex mathematical equation (Hackman 2011). The field of personnel psychology was at a similar juncture in the early 1980s when individual occupational performance was thought to be situationally specific. This Theory of Situation Specific Validity (TSSV) meant that “A test valid for a job in one organization or setting may be invalid for the same job in another organization or setting” (Schmidt and Hunter 1981, p. 1132). Practically, the TSSV suggested occupational success was contingent upon how one’s idiosyncratic attributes fit the job-type and unique aspects of the situation—which arguably reflects current sentiments in the field of group performance (see Hollenbeck et al. 2012).

The edifice of the TSSV as it relates to individuals was found to be methodologically flawed and was systematically dismantled by Schmidt and Hunter’s application of the theory of general intelligence to occupational performance where IQ tests have proven highly valid for explaining variance in individual performance irrespective of the job type or situation (Schmidt and Hunter 1981,1998, 2004). The most recent iteration of the operational validity of general intelligence and occupational performance remains, averaged across context and job type, relatively large (*r* = .65) (Schmidt et al. 2016).

If the claims of Woolley et al. (2010) are correct in that a domain-general factor exists in groups as in individuals, one may expect a similar dismantling of the majority view such that a *c-*factor supersedes the importance of situationally specific factors, tasks, team types, and contexts governing group performance. If the *c-*factor proves to be a valid construct, it may suggest that groups exhibit high levels of intelligence (i.e., “smart groups”) even when individual members lack these attributes (and the reverse may also hold). Selecting for and cultivating high levels of *c*, unlike situation-specific approaches or those which rely on the attributes of individual members (e.g., individual intelligence), would maximize gains because benefits to group performance would be reaped across multiple rather than isolated domains and contexts—rendering organizational groups more capable of exploiting opportunities and weathering disruptions. Traditional entry and selection practices, especially those predicated on identifying individuals with the attributes necessary to optimize group performance (e.g., Barrick et al. 1998), would necessarily fall by the wayside in favor of selecting for and promoting the attributes of effective groups. The potential implications for the existence of the *c-*factor can hardly be overstated.

Yet few studies have sought to replicate the *c-*factor. The present investigation found one published attempt to compare and contrast results from a sample of six available empirical studies exploring the validity of the *c-*factor (Credé and Howardson 2017a, b). It involved re-analyzing results from six independent samples (*K* = 6) across four studies and concluded that the data does “not support the inference that a general factor can explain substantial variation in performance across a wide cross section of group tasks” (p. 1491). However, Credé and Howardson (2017a, b) did not consider the effect of the *c-*factor on criterion-relevant tasks outside of and subsequent to the group-IQ testing battery. Moreover, research examining the relative validity of the *c-*factor compared to the *g-*factor in group performance settings is limited. Therefore, an opportunity exists to build on the extant literature addressing the validity of the *c-*factor and meta-analyze the relative impact both the *c-*factor and *g-*factor have on outcomes external to the battery of tests used to derive these predictor variables. We attempt to answer the following questions:

- Question 1: What evidence supports the validity of the *c-*factor and its effect on group performance?
- Question 2: What evidence distinguishes Spearman’s *g-*factor from Woolley et al.’s *c-*factor?

To achieve this, we seek to review empirical studies that directly explore the validity and effects of the *c-*factor. We preference quantitative data from studies that have employed methods and measures (i.e., the group IQ paradigm) similar to those outlined in the original study by Woolley et al. (2010).

## Method

### Inclusion/exclusion criteria

Included within this review are studies that: attempt to directly or conceptually replicate a collective intelligence factor (i.e., the *c-*factor) in the broader context of its effect on group performance; completed data collection and reporting between the initial publication of Woolley et al. in September 2010 and November 2019—including published or unpublished reports. Articles/reports were *excluded* if: quantitative results were not available by publication, pre-print, or inspection by personal request (e.g., Jones 2015); they were based on simulation rather than data representing real humans (e.g., Chmait et al. 2016; De Vincenzo et al. 2018); results were primarily conceptual and/or qualitative such as case studies, ethnographies, essays, or conceptual reviews (e.g., Gunasekaran et al. 2016; Krafft 2018; Nagar 2016; Runsten 2017; Salminen 2012); full text was not available in English (e.g., Del Cerro et al. 2016); used empirical methods, such as a survey or questionnaire, but failed to employ the group IQ testing approach outlined in Woolley et al. (2010) (e.g., Kaur and Shah 2018; Lee and Jin 2019); failed to explicitly test for the existence of the *c-*factor and/or its effect on group performance (e.g., Hansen and Vaagen 2016).

## Search methods and yield

**Fig. 1:**
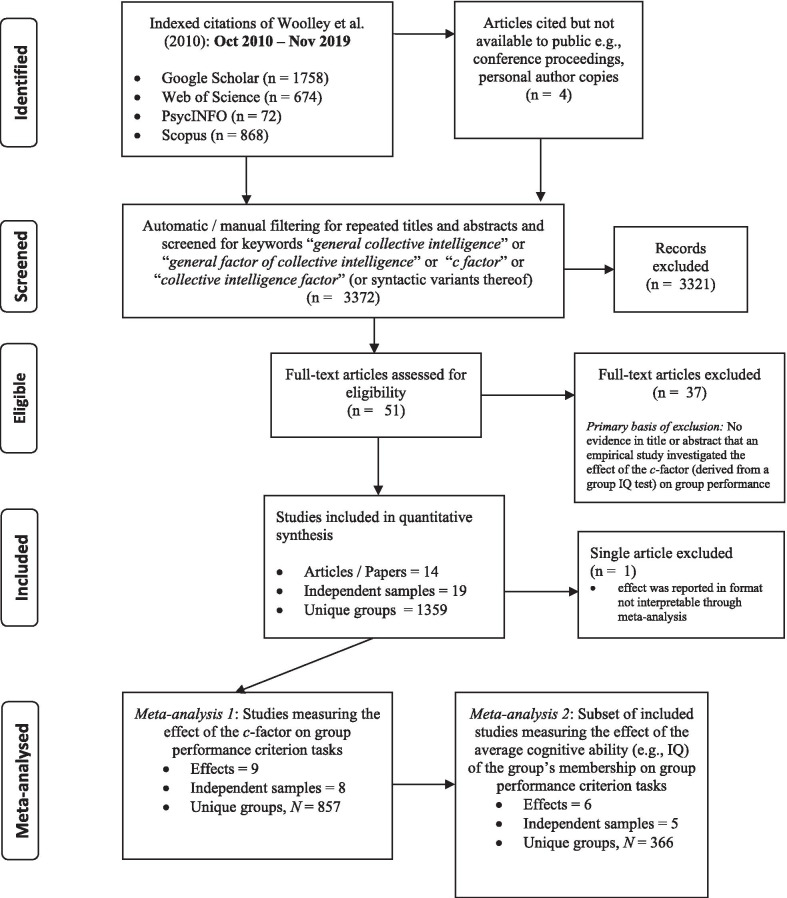
Flow Diagram for Study Inclusion/Exclusion. *Note.* Citations were searched within indexed citations of the original article by Woolley et al. (2010) “Evidence for a Collective Intelligence Factor in the Performance of Human Groups”

We initially searched articles that directly cited Woolley et al. (2010) on Google scholar, Web of Science, PsycINFO, and Scopus between the time of the original study (October 2010) and September 2018. An updated search was conducted in preparation for this journal using the same process and databases outlined above, with the exception that publication dates were restricted to articles published between January 2018 and November 2019. Searches were conducted inspecting titles and/or abstracts containing the term “collective intelligence factor” (or syntactic and semantic variants thereof, e.g., general factor of collective intelligence, *c-*factor). Articles were flagged for full-text review if they clearly met or had the potential to meet the inclusion criteria (and did not violate the exclusion criteria). Figure 1 represents the flow diagram for combined (initial and updated) searches. All datasets, scripts generated and/or analyzed during the current study, and the two (uncombined) flow processes are available in the OSF repository, https://osf.io/xevkj/.

## Results

### Qualitative results

Overall, our search yielded fourteen separate studies, involving data from 19 independent samples comprising 1359 groups ranging from 2 to 6 members in size. Most samples (11 of 18) were exclusively located in the USA, based in laboratories (14 of 19), completed a group IQ test battery involving an average of 6.3 subtests, and did so either partially (2 of 19) or fully (12 of 19) online—with the remainder (5 of 19) completed by face-to-face (F2F) groups. Most studies employed correlational designs in which random allocation was used (13 of 18 samples and one not reported), although studies rarely reported randomizing the order of subtest administration—thus failing to account for order effects and between-test dependences. Only 7 of 19 samples were independent of Woolley and affiliated co-authors. All attempts to reproduce the *c-*factor under independent authorship either partially or completely failed, while all Woolley-affiliated attempts to reproduce the *c-*factor succeeded. Also included, for the purposes of qualitative and secondary analysis, were two reports containing secondary data (Bates and Gupta 2017; Credé and Howardson 2017a, b). A summary of results can be seen in Table 1. Finally, email request was made in September 2018 for access to results/data from a pre-registered experiment by Gimpel and Graf; however, no results were shared as data collection remained ongoing (Gimpel et al. 2018). Having fully inspected the pre-print, this study was flagged for future inclusion.

**Table 1:** Summary of Empirical Studies on Collective Intelligence

Study name	*N*	*K*	Effect 1: % Var	Effect 2: (%) Pos. Manifold	Effect 3: c → criterion (*r*)	Effect 4: Av.IQ → c (*r*)	Effect 5: Av.IQ → criterion (*r*)	RA (Y:N)
^a^Credé and Howardson (2017a, b)	*487*	*6*						5:1
^a^Bates and Gupta (2017): Studies 2 and 3 (combined analysis)	*80*	*2*						Yes
Woolley et al. (2010, ): Study 1	40		43.4	100	.52	.19, p = ns	.18, p = ns	Yes
Woolley et al. (2010, : Study 2	152		44.1	93	.28	.15, p = .04	.18, p = ns	Yes
Engel, Woolley, Jing, Chabris, and Malone (, 2014a, b): Face-to-face (speaking) condition	32		49.3	100				Yes
Engel, Woolley, Jing, Chabris, and Malone (, 2014a, b): Online (text-chat) condition	36		41.4	100				Yes
Engel et al. (2015a, b): Study 2	116		40	100	.25			No
Engel, Woolley, et al. (2015a, b): Study 3	25		40	100				Yes
Woolley & Aggarwal (under review); (Also reported in Woolley and Aggarwa^2017^)	59				C1: .29 and C2: .29	-.05, p = 53	C1: -.02, p > .05; C2: -.21, p > .05	No
Meslec et al. (2016)	30							Yes
Glikson, Harush, et al. (under review)	115				.11			No
Chikersal, et al. (2017)	58							No
Kim et al. (2017a, 2017b)	248		38.38		-.15			Yes
Aggarwal et al. (2019)^d^	98		44		.58^e^			Yes
Barlow and Dennis (2016, )	86		42	50			.07 (p > .05)	Yes
Barlow (2015, unpublished doctoral thesis): Control Group (CG)	64			33				Yes
Barlow (2015, unpublished doctoral thesis): Experimental Group (EG)	65		46	100		.339, p = 026 ^c^		Yes
Bates and Gupta (2017): Study 1	26		39.8	100				Yes
Bates and Gupta (2017): Study 2	40		50	100				Yes
Bates and Gupta (2017): Study 3	40			100				Yes
Rowe (2019, unpublished doctoral thesis)	29		41	100	.104, p = .59	.294, p = .12	.202, p = .29	No
Mean or *Ratio*:	71.5		43.03	90	.253	.185	.067	13:5

Study name	Subtests (n)	Independent: Woolley	Lab: Field	Online or Face-to-face ^b^	Group size	Country (USA:Other)
^a^Credé and Howardson (2017a, b)	≥ *4* < *17*	*Yes*	*Both*	*Both*		*Multiple*
^a^Bates and Gupta (2017): Studies 2 and 3 (combined analysis)	5	Yes	Lab	*Both*	3	*Multiple*
Woolley et al. (2010, ): Study 1	5	No	Lab	F2F	3	USA
Woolley et al. (2010, ): Study 2	10	No	Lab	F2F	2 to 5	USA
Engel et al. (2014a, b): Face-to-face (speaking) condition	8	No	Lab	F2F	4	USA
Engel et al. (2014a, b): Online (text-chat) condition	8	No	Lab	online	4	USA
Engel et al. (2015a, b): Study 2	7	No	Field	online	2 to 5	Germany
Engel et al. (2015a, b): Study 3	6	No	Lab	online	4	Japan
Woolley and Aggarwal (under review); (Also reported in Woolley & Aggarwal, 2017)	8	No	Field	online	4 to 5	USA
Meslec et al. (2016)	8	No	Field	online	3 to 6	Netherlands
Glikson, Harush, et al. (under review)	8	No	Field	online		USA
Chikersal, et al. (2017)	6	No	Lab	online	2	USA
Kim et al. (2017a, b)	11	No	Field	online	5	Multiple
Aggarwal et al. (2019)^d^	5 or 10	No	Lab	Both	2 to 5	USA
Barlow and Dennis (2016, )	3	Yes	Lab	online	3 to 5	USA
Barlow (2015, unpublished doctoral thesis): Control Group (CG)	3	Yes	Lab	online	3 to 5	USA
Barlow (2015, unpublished doctoral thesis): Experimental Group (EG)	3	Yes	Lab	online	3 to 5	USA
Bates and Gupta (2017): Study 1	5	Yes	Lab	F2F	2 to 4	UK
Bates and Gupta (2017): Study 2	5	Yes	Lab	Both	3	India
Bates and Gupta (2017): Study 3	5	Yes	Lab	Both	3	UK
Rowe (2019, unpublished doctoral thesis)	5	Yes	Lab	F2F	2 to 5	Australia
Mean or *Ratio*:	6.3	*7:12*	14:5		na	*11:8*

*Note*: The table outlines empirical studies on collective intelligence and group performance published between October 2010 and November 2019. *n* = number of groups. *K* = number of independent samples. Effect 1 = Percentage of total variance in group IQ composite explained by first factor/component; Effect 2 = The proportion of positive correlations within the correlation matrix comprised of bivariate Pearson's correlations, *r,* between group IQ test items (positive manifold test); Effect 3 = Bivariate Pearson's correlation, *r*, between *c* and a criterion task; Effect 4 = Bivariate Pearson's correlation, *r*, between Av.IQ and *c*; Effect 5 = Bivariate Pearson's correlation, *r*, between Av.IQ and Criterion task; ns = *p* > .05; RA = Random Allocation to groups; F2F = Face-to-face. C1 and C2 = Criterion task 1 and 2 of a single study; *r* = Pearson’s correlation coefficient. Mean and Ratio scores include only primary data and therefore exclude previously meta-synthesized results from Credé and Howardson ( 2017a, 2017b), and Bates and Gupta (2017): Studies 2 and 3 (combined)

^*a*^*Pooled data from secondary sources (*≥ *2 studies) not included in the present analysis.*

^b^ This pertains only to group IQ testing context and not to the group performance setting

^c^Correlation exists for the EG only (the *c-*factor was not apparent in the CG)

^d^Paper originally added as a conference proceeding (Aggarwal & Woolley, 2014)

^e^Result was originally reported in *R*^2^ value, controlling for intercept and team size, then transformed to a correlation coefficient using square root(.34) = .58 (see Aggarwal et al. 2019, p. 6)

## Quantitative results

The nature of our research questions meant that results could not be synthesized into a single effect. We instead decided (post hoc) to sort results into five different categories based on the effect they quantified:

- *Effect 1:* The percentage of variance in group IQ tests scores explained by the first factor/component following exploratory factor analysis (EFA);
- *Effect 2:* The presence of a positive manifold, including the distribution of positive bivariate correlations between group IQ test items;
- *Effect 3:* Bivariate Pearson's correlation, *r*, between the *c-*factor and a criterion task external and subsequent to the original group IQ test battery;
- *Effect 4:* Bivariate Pearson's correlation, *r*, between the average IQ of the group’s individual members and the group’s regression loading scores from the *c-*factor;
- *Effect 5:* Bivariate Pearson's correlation, *r*, between the average IQ of the group’s individual members and a criterion task external and subsequent to the group IQ test battery.

### Effect 1: the c-factor

For effect 1, the average variance between group IQ test battery scores explained by the first factor (or principal component) extracted via EFA was 43% (*SD* = 3.6%). The largest value was reported in the second experiment by Bates and Gupta (2017) at around 50%. They interpreted this factor as directly related to the *g*-factor possessed by and manifest in the groups’ individual members, suggesting the *c-*factor need not be invoked to explain the results.

One reviewer expressed concern about the validity of this effect in the context of common factor analysis because the percentage of common variance explained by the first factor can remain high even when covariation among group IQ test items is neither uniformly strong nor general. While we are sympathetic to the reviewer’s point that the total variance explained by the initial factor (or component) is necessary but insufficient to establish validity, we believe this concern is allayed by close inspection of the correlation matrix. We note that most studies report the total variance explained by the initial eigenvalues, which combines common and unique (specific and error) variance from the IQ composite. The original study by Woolley et al. (2010), for example, established a priori that the first factor must account “for 30 to 50% of the [total] variance” (p.687) among a diverse set of cognitive tasks. This was later confirmed by Woolley et al. in both studies 1 and 2, with the initial factor accounting for 43% and 44% of the (total) variance, respectively. This was the most frequently reported statistic among studies herein and recognized as central to the evidence used in considering the internal validity of the hypothesized *c-*factor. The relative magnitude of this statistic also provided the basis for rejecting the *c-*factor in at least one study (e.g., Barlow and Dennis 2016). Therefore, reporting this statistic is vital for explaining and comparing the relative roles played by *g* and *c* in the test composite as long as it is considered alongside other findings (especially effect 2) and in the broader theoretical context of this review.

The same reviewer suggested using the average variance extracted (AVE %), which provides an index of the “generality” of a general factor in terms of how well it explains the percentage of covariation across a range of tasks relative to measurement error. In the case of the studies reporting on the *c*-factor, the AVE can be calculated by taking the average of the squared standardized loadings between the *c*-factor and each of the group tasks used to substantiate the group IQ test composite as displayed in Table 2.

**Table 2:** Standardized loadings and average variance extracted across 8 samples

Group IQ subtest	Woolley et al. (2010 , Sample 1)	Woolley et al. (2010 , Sample 2)	Engel et al. ( [Bibr CR35][Bibr CR126])	Engel et al. ( [Bibr CR34][Bibr CR125], Sample 1)	Engel et al. ( [Bibr CR34][Bibr CR125], Sample 2)	Barlow and Dennis (2014)^a^	Bates and Gupta 2017 (Samples 2 and 3 combined)^b^	Rowe (2019)
Brainstorming	.32	.58	.7	.7	1	1	.38	.57
Matrix Reasoning	.73	.61	.72	.47	.43		.74	.48
Moral reasoning	.36	.11				-.25	.62	
Plan shopping trip	.57	.23					.48	
Typing	.69	.48	.67	.71	0		.72	
Word completion (Beginning with)		.75						
Spatial problems		.47						
Incomplete words (Missing letters)		.47						
Estimation problem		.32						
Reproducing art		.34						
Unscramble words			.57	.57	.4			
Sodoku			.61					
Judgment tasks			.37	.3				
Memory			.56	.65	.26			.92
Detection			.43	.33	.52			
Decision						-.14		
Mill Hill vocabulary								.24
Multiple choice vocabulary (synonyms)								.14
AVE (%)	31.32	22.26	34.87	30.88	28.05	36.07	36.50	29.58

*Note.* Results display those reported across 8 samples (Bates and Gupta 2017 is in combined form) and indicate the standardized loadings of the *c*-factor onto the respective subtest. AVE = Average variance extracted based on the statistical average of the squared loadings from each of the subtest results in the samples listed above

^a^The standardized loading from the “complex task” was not included in this table because it was used as an external (predictive) validity criterion

^b^These standardized loadings are taken from a multilevel structural equation model that combined data from the subtests used across studies 2 and 3 in Bates and Gupta (2017, p.53)

The AVE is 30.23% when calculated across all 49 standardized loadings for each subtest and ranges from 22 (Woolley et al. 2010 , sample 2) to 37% (Bates and Gupta 2017, samples 2 and 3 combined). These values fall substantially below the minimum 50% recommendation advocated by some authors (e.g., Fornell and Larcker 1981; Hair et al. 2014), which requires that the *c*-factor shares an average loading with its indicators of .71; an effect that, if partitioned into a univariate model, would equate to a “very strong association” (see Rosenthal 1996 for rationale and equations). The justification for the “50% AVE” cutoff, however, does not stem from empirical findings but instead from the idea that a factor and its indicators are “questionable” when variance explained by measurement error exceeds that explained by the purported factor in relation to its indicators (Fornell and Larcker 1981, p.46). A more recent review by Credé and Harms (2015), while acknowledging the AVE 50% cutoff as respectable, suggests such thresholds should be interpreted in the broader empirical context of the field and used alongside model fit indices to gauge the validity (or lack thereof) of a purported general factor.

It is worth noting that the *g*-factor, despite being touted as the “holy grail” of human abilities (see McGrew 2009), may fail to meet such expectations in the context of the studies included in this review. For example, in the study by Rowe (2019), 85 participants completed the ICAR-16, an IQ test containing four subtests (matrix reasoning, letter-number reasoning, verbal reasoning, and 3D rotation), and established as a part of a broader open-science project by the International Cognitive Ability Resource (ICAR) team (Condon and Revelle 2014; Dworak et al. 2020). The resulting single-factor model (i.e., the *g*-factor model) obtained from the CFA displayed excellent model fit (*χ*^2^ (2) = 0.932, CFI = 1.0, TLI = 1.11, RMSEA = .00, *p* = .628) and yet the AVE was 30% which, as is the case with the AVE values obtained from the *c*-factor analyses in Table 2, falls well below the 50% recommended cutoff. If such cutoffs are to be taken seriously, they would also likely call into question a substantial proportion of the hundreds of datasets found in the Human Cognitive Abilities (HCA) Dataset Archive (http://www.iapsych.com/wmfhcaarchive/wmfhcaindex.html) and used to establish what is arguably the most widely endorsed theory of general intelligence, the CHC theory (outlined in detail in McGrew 2009). Therefore, in addition to the AVE and model fit indices, it is imperative to consider other available empirical and theoretical information when evaluating the validity of the hypothesized *c*-factor.

### Effect 2: positive manifold

Effect 2 examined the presence of a positive manifold. A positive manifold is observed when bivariate correlations between test items in the correlation matrix are positive (Jensen 1998, p. 24; Jensen and Weng 1994, p. 246). Worth considering is that correlation matrices among intelligence test items rarely display universally positive and universally high coefficients between test scores. Instead, low loadings and negative loadings are frequently encountered. The HCA Dataset Archive (mentioned in the preceding section) hosts hundreds of correlation matrices from nearly as many IQ tests. Close inspection of these matrices often reveals low, zero, and negative correlations among items that otherwise tend to positively correlate. Crucially, these patterns did not prohibit Carroll (1993) from devising what is arguably *the* putative theory of general intelligence: the Cattell-Horn-Carroll (CHC) theory (see Schneider and McGrew 2018). Rather than considering the violation of the positive manifold incompatible with a general factor, it may instead indicate that uniquely tainted items, the proverbial “rotten apples,” should be removed from subsequent iterations of the test. Thus, a general factor may exist even if it is not indifferent to all indicators but common only to a subset of them. The search for a positive manifold in the correlation matrix should consequently be approached with some degree of leniency toward rogue items that would otherwise “spoil the bunch.” In the present inquiry, we examined all available correlation matrices for group IQ subtests across 12 samples (primary studies only). This involved recording the frequency of bivariate correlations within a specified range. For example, if a correlation matrix had three values .11, .14, and .19, a value of “3” would be entered between .10 and .20 for that study, as seen in Table 3.

**Table 3:** Counts of correlations in specified ranges for item matrices across 12 studies

Correlation range:	−.20 to −.30	−.10 to −.20	.0 to −.10	0 to .10	.10 to .20	.20 to .30	.30 to .40	.40 to .50	.50 to .60	.60 to .70	.70 to .80	.80 to .90	.90 to 1.0	Count (total)	Weight (%)	% Positive
Woolley et al. (2010): Study 1					3	3	2	1	1					10	5.85	100
Woolley et al. (2010): Study 2			3	12	13	8	5	4						45	26.32	93.33
Engel, Woolley, Jing, Chabris, and Malone (2014a): Combined				1	1	11	4	8	3					28	16.37	100
Engel, Woolley, et al. (2015a): Japan				4	3	4	1	2	1					15	8.77	100
Engel, Woolley, et al. (2015): Germany				2	5	6	1	7						21	12.28	100
Barlow and Dennis (2016)	1	1	1	2	1									6	3.51	50
Barlow (2015, unpublished doctoral thesis): Control Group (CG)	1	1				1								3	1.75	33.33
Barlow (2015, unpublished doctoral thesis): Experimental Group (EG)					2	1								3	1.75	100
Bates and Gupta (2017): Study 1					2		4	2	1		1			10	5.85	100
Bates and Gupta (2017): Study 2						2	2	1	2	2		1		10	5.85	100
Bates and Gupta (2017): Study 3				2	5	1	1			1				10	5.85	100
Rowe (2019, unpublished doctoral thesis): Study 1				2	3	1	2	1	1					10	5.85	100
Total Count:	2	2	4	25	38	38	22	26	9	3	1	1	0	171	100.00	
Percentage (%)	1.17	1.17	2.34	14.62	22.22	22.22	12.87	15.20	5.26	1.75	0.58	0.58	0.00			

*Note*: Numbers represent counts of bivariate correlations within a specified range for a given study. Count (total) displays the total number of correlations per study (rows). Weight (%) refers to the proportion of the total number of correlations (N = 171) analyzed. % Positive calculates the proportion of bivariate correlations that are positive per study (rows). CG = Control group. EG = Experimental group

**Fig. 2:**
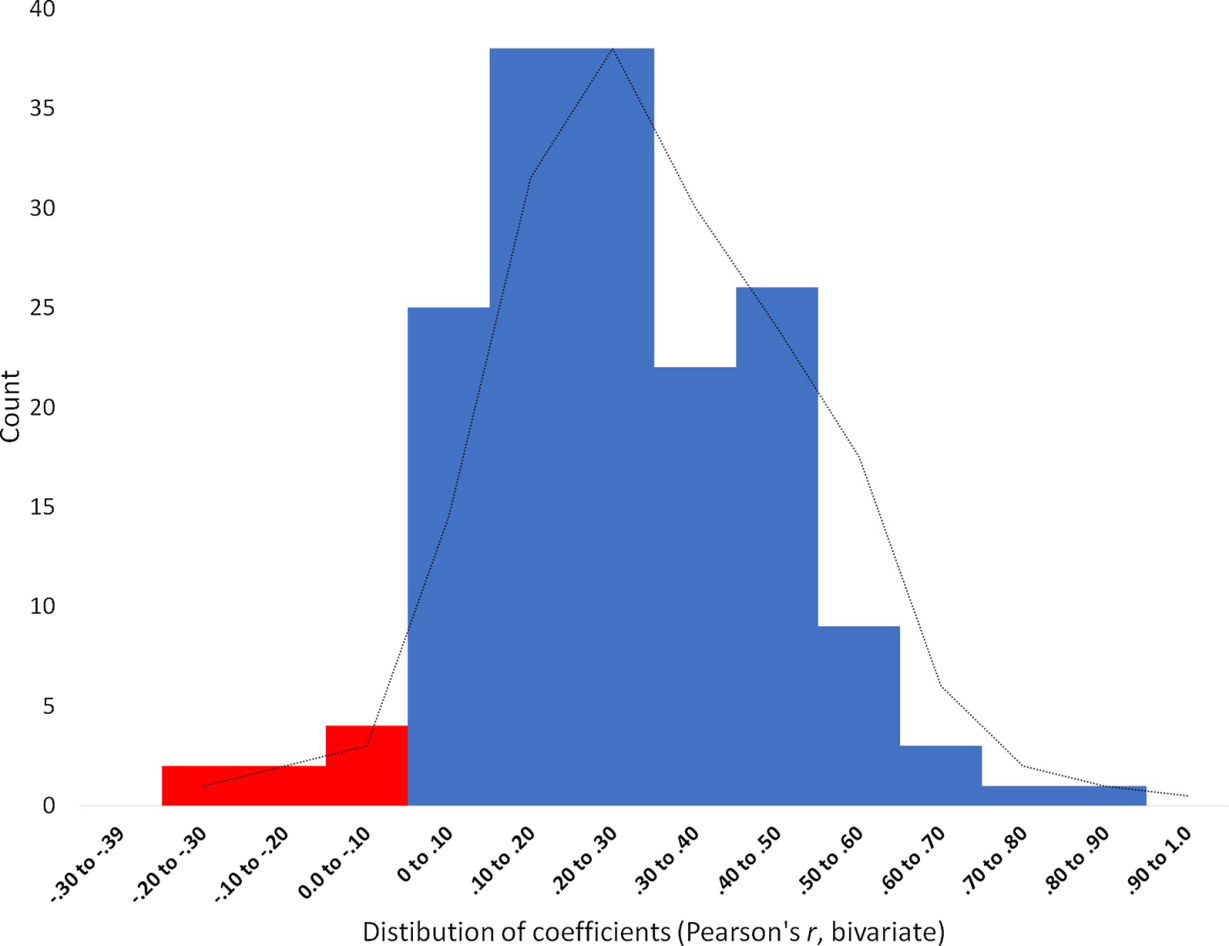
Histogram of the Frequency (Count) of Distributions of Bivariate Correlations Between Group IQ Items (within study). *Note.* The frequency of negative and positive correlations between group IQ test items is represented by red and blue bars, respectively. The dotted line –- represents the trendline for the average frequency

Most striking is that 95% of correlations between group IQ test items scores within each study are positive (163 out of 171), with an obvious positive skew (see Fig. 2): 59% were low to moderate (0 to .30), 28% moderate to large (.30 to .50), and 8% were large to extra large (.50 or greater) in approximation with effect size conventions set out by Cohen (1988).

Extremely low and negative correlations were mostly attributable to the studies by Barlow (2015) and Barlow and Dennis (2016). These studies included a brainstorming task (e.g., Think of as many ideas as you can to increase tourism in your local town), a college admissions task (e.g., Out of four candidates, decide which are the two best prospects to admit to the university), and a planned shopping trip task (e.g., plan a shopping trip by deciding which stores to visit to purchase items from a list). A fourth task, the candy production firm, was used for “robustness checking” and required groups to maximize the profits of a candy production firm by optimizing the distribution of ingredients. It was selected as a criterion task on the grounds that it was more complex because it encompassed qualitative features of the other three tasks. However, this was a problematic assumption because correlations were .02, .01, and .15 between the “complex task” and the brainstorming, college admission, and shopping trip, respectively. These results are somewhat unsurprising considering evidence of the psychometric reliability, and validity of these tasks in IQ testing environments is lacking. In contrast, some of the strongest pairwise correlations among group IQ test times were found in the three studies by Bates and Gupta (2017) and, in particular, between items known to load heavily onto psychometric *g* (e.g., Ekstrom et al. 1976; Raven 2000). Missing letters, for example, correlated strongly with word fluency (*r* =  .80) and Raven’s advanced progressive matrices (*r* = .64).

Therefore, despite most correlations among items in the correlation matrices being positive, the strength of these collinearities was highly variable. Items that had previously been validated for IQ testing tended to share the strongest correlations, while items with low face validity for IQ testing tended to share lower and/or negative correlations.

### Effect 3: the c-factor and criterion performance

For effect 3, nine separate criterion tasks pertaining to eight independent samples (*K* = 8, *N* = 857) were included with the aim of measuring the effect of the *c-*factor on a group performance criterion task external to the group IQ test battery. A brief description of the criterion tasks used across these studies can be seen in Table 4. A notable characteristic of most of the tasks is that they were often conducted in laboratory contexts and/or with the use of relatively contrived group tasks that may limit inferences about the real-world predictive validity of the *c*-factor.

**Table 4:** External criterion tasks

Study	Criterion	Description
Woolley et al. (2010): Study 1	Computerized checkers	A group sat in front of a single screen, were trained for 5 min, and played a single match of checkers against a computerized opponent
Woolley et al. (2010): Study 2	Architectural design task	Groups design and build a house, garage, and pool with limited materials and strict building codes. (10 min planning, 20 min building)
Enge et al. (2015a, b): Study 2 (Germany)	Student project	Student team projects were completed and rated by university students (peer review)
Woolley and Aggarwal (under review); (Also reported in Woolley and Aggarwal 2017)	Group learning	Slope (rate and size) of learning gains in 4 × repeated MBA student exams over 6 weeks
Woolley and Aggarwal (under review); (Also reported in Woolley and Aggarwal 2017)	Group synergy	As above. Synergy slope was measured against coordination and process gains attributable to groups once individual gains are controlled for on the slope
Glikson, Harush, et al. (under review); also reported in Woolley, Glikson, Haan, Harush, and Kim, (2018)	Group presentation	Student group PowerPoint presentation worth 40–60% of final subject score (establish new business in foreign country), measured with significant (e.g., semester) delay post group IQ test
Kim et al. ([Bibr CR60][Bibr CR128])	Group learning	'League of Legends' video-game team learning behavior (via Edmondson’s scale of error detection / correction); repeated measure T1 = baseline and T2 = 6-month follow-up (learning = T2-T1)
Aggarwal et al. (2019)	Group learning	Group learning defined as “the rate of change (or slope) in earnings for each group across ten rounds of the [minimum-effort tacit coordination] game” (a behavioral economics game, see p.5). Results controlled for team size and intercept
Rowe (2019)	Group decision-making and prioritization task (moon survival)	A hypothetical situation in which a crew stranded on the moon must survive the journey back to their mothership with only 15-items salvaged from the wreck of their explorer craft. Items must be ranked according to their survival utility and compared against experts. (6 min)

*Note*: Criterion tasks were always measured external and subsequent to the group IQ test battery; an additional study by Engel et al. ([Bibr CR35][Bibr CR126]) used the Desert Survival Task or DST (as reported in study one of Engel et al. [Bibr CR34][Bibr CR125]) as the criterion and reported an outcome of *b* = .24 and *p* = .058. The DST asks groups to rank, in order of survival value, a random set of items while stranded in a desert. Results were not included in the meta-analysis because we were not clear about: (a) whether the beta coefficient was (un)standardized; (b) what the predictor variable was (it was assumed to pertain to the regression weight of the *c-*factor); (c) and was not reported as to whether the score pertained to the online, face-to-face, or combined subset of the sample

**Fig. 3:**
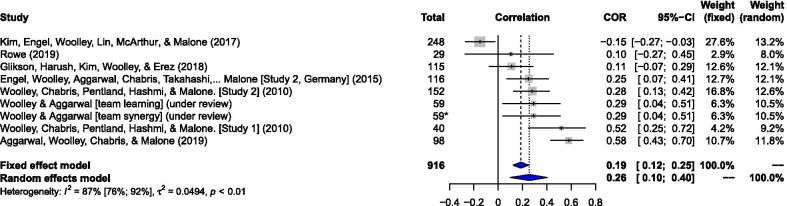
Forest plot: The *c-*factor and Criterion Tasks. Note. Results are displayed for a random effects model pertaining to correlations between the c-factor and various criterion tasks. The c-factor scores are operationalized using factor/principal component regression weights. Most studies did not report which method was used to calculate these weights (e.g., Bartlett method). All studies included in this analysis, except for Rowe (2019), were undertaken by Woolley and colleagues and therefore employed procedures and measures described in Woolley et al. (2010) and/or Engel et al. ( [Bibr CR35][Bibr CR126]). Criterion tasks were different across all studies. Box sizes are relative to sample weights. *Two effects (team learning and team synergy) by Woolley and Aggarwal (under review) from one unique sample (n = 59) were included in this meta-analysis, leading to a total N = 916 when the sample was added on the basis of each unique effect or a total N = 857 when the sample was added on the basis of each unique group

The Metafor package in RStudio was used to meta-analyze correlations between the *c-*factor and group performance on the criterion tasks (Viechtbauer 2010). We employed a random effects model using the restricted maximum likelihood estimator function. This model revealed a small to moderate sample weighted correlation, *r*, of .26 (95% CI .10, .40). Results were widely dispersed and revealed a significant Cochran’s *Q*(8) = 59.36, *p* < .001, and heterogeneity between effects was high (*I*^2^ = 86.5%, Tau^2^ = .05). Results were also replicated using Comprehensive Meta-analysis software version 3 (Borenstein et al. 2014). A summary of these findings, including a forest plot, can be seen in Fig. 3.

**Fig. 4:**
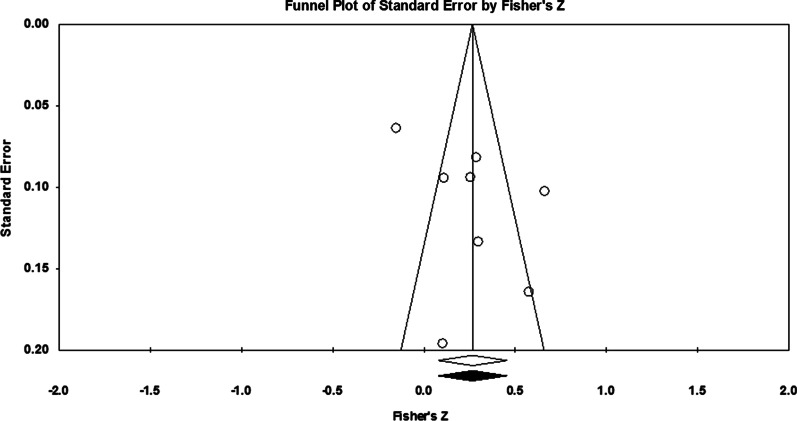
Funnel plot: Publication Bias and the *c-*factor. Note. The funnel plot estimates the number of missing studies, assuming they retain the null (e.g., demonstrate zero correlation), required to reduce the Z-value below a .05 alpha (two-tailed) cutoff of 1.96. In this instance, the presence of empty dots (i.e., actual studies) and the absence of black-filled dots (i.e., imputed studies) suggest publication bias has not been detected using this test

Publication bias estimates can be visually inspected via the actual and imputed results in the funnel plot (see Fig. 4), which suggest no missing studies in this analysis. Results, however, should be interpreted alongside other tests for bias as it lacked the recommended number of studies (*k* ≥ 10) to readily gauge asymmetries (Sterne et al. 2011). The Classic fail-safe *N* test (i.e., file-drawer test) suggests that 96 studies with null (zero) correlations would be required to nullify the point estimate (mean) *Z*-value of 6.66 to 0, assuming an alpha of .05 (*Z*-value of 1.96). This coheres with Rosenthal’s tolerance level for (5* k* + 10) which, in this instance, would estimate no more than 45 studies in the file drawers for this dataset (Rosenthal 1979, p. 640). Egger’s linear regression intercept did not show any significant indication of publication bias (*p* = .124).

### Effect 4: The c-factor and the g-factor

Effect 4 examined the correlation between the average IQ of the group and the *c-*factor derived from regression weights of the first factor or principal component. This metric was reported for five independent samples (*K* = 5, *n* = 345 groups). The bivariate Pearson’s correlation between average IQ and the *c*-factor was .19, a rather surprising result considering these parameters were estimated from vastly different items (e.g., Which shape best completes the geometric pattern? vs. Which applicant should be admitted as a student to the university?). The two studies *independent* of Woolley and affiliates showed an average correlation between average IQ and the *c-*factor of .32, while the two studies (comprising three samples) by Woolley and affiliates showed an average correlation of .10. The relationship between the average IQ of the group’s individual members and the regression weights derived from the *c-*factor was, overall, small to moderate, and this association seemed to be larger when considered apart from the studies by Woolley et al. This tentatively implies the *g-*factor and the *c-*factor may only be marginally overlapping constructs, but such conclusions should be tempered by several important methodological considerations (see “Discussion” section).

### Effect 5: The g-factor and criterion performance

**Fig. 5:**
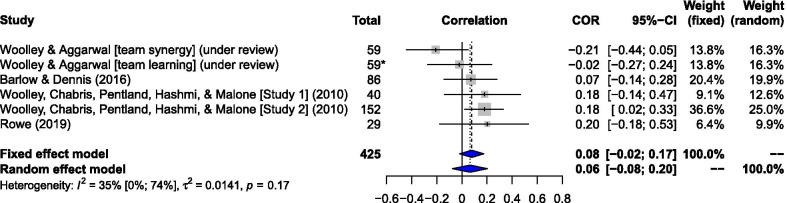
Forest plot: Average IQ and Criterion Task Performance. *Note.* Results are displayed for a meta-analysis using a random effects model for correlations between average IQ scores and group performance on criterion tasks. The average IQ is operationalized using the total IQ scores of each individual member divided by the number of members (= ∑IQ / n). The study by Barlow and Dennis (2016) did not apply the c-factor to the criterion task (the profit maximization task) because the authors found it was not valid. Studies behind the other (five) correlations, however, did apply both the c-factor and Av.IQ to the same criterion task—allowing for relative comparison. The Wonderlic Personnel Test (WPT) was used for all except Woolley et al. (2010) Study 1 where 18 odd items of 36-item Raven’s Advanced Progressive Matrices were used and in Rowe (2019) where the ICAR-16 was used. Box sizes are relative to sample size weights. *Two effects (team learning and team synergy) by Woolley and Aggarwal (under review) from one unique sample (n = 59) were included in this meta-analysis, leading to a total N = 425 when the sample was added on the basis of each unique effect or a total N = 366 when the sample was added on the basis of each unique group

Using a random effects model*,* five independent samples (*K* = 5, *N* = 366 groups) with six effects actively controlled for the influence of the *g-*factor using the statistical mean IQ of individual members (see Fig. 5). A sample weighted correlation showed little to no discernible relationship with group performance on the criterion tasks (*r* =  .06), although much of the distribution lies above zero (95% CI −.08, .20) suggesting the population-level effect is more likely to reflect this. The average score of the raw correlations was *r* = .07. Heterogeneity was moderate (*I*^2^ = 35%, Tau^2^ = .014) but not statistically significant according to Cochran’s *Q*(5) = 7.72, *p* = .17.

**Fig. 6:**
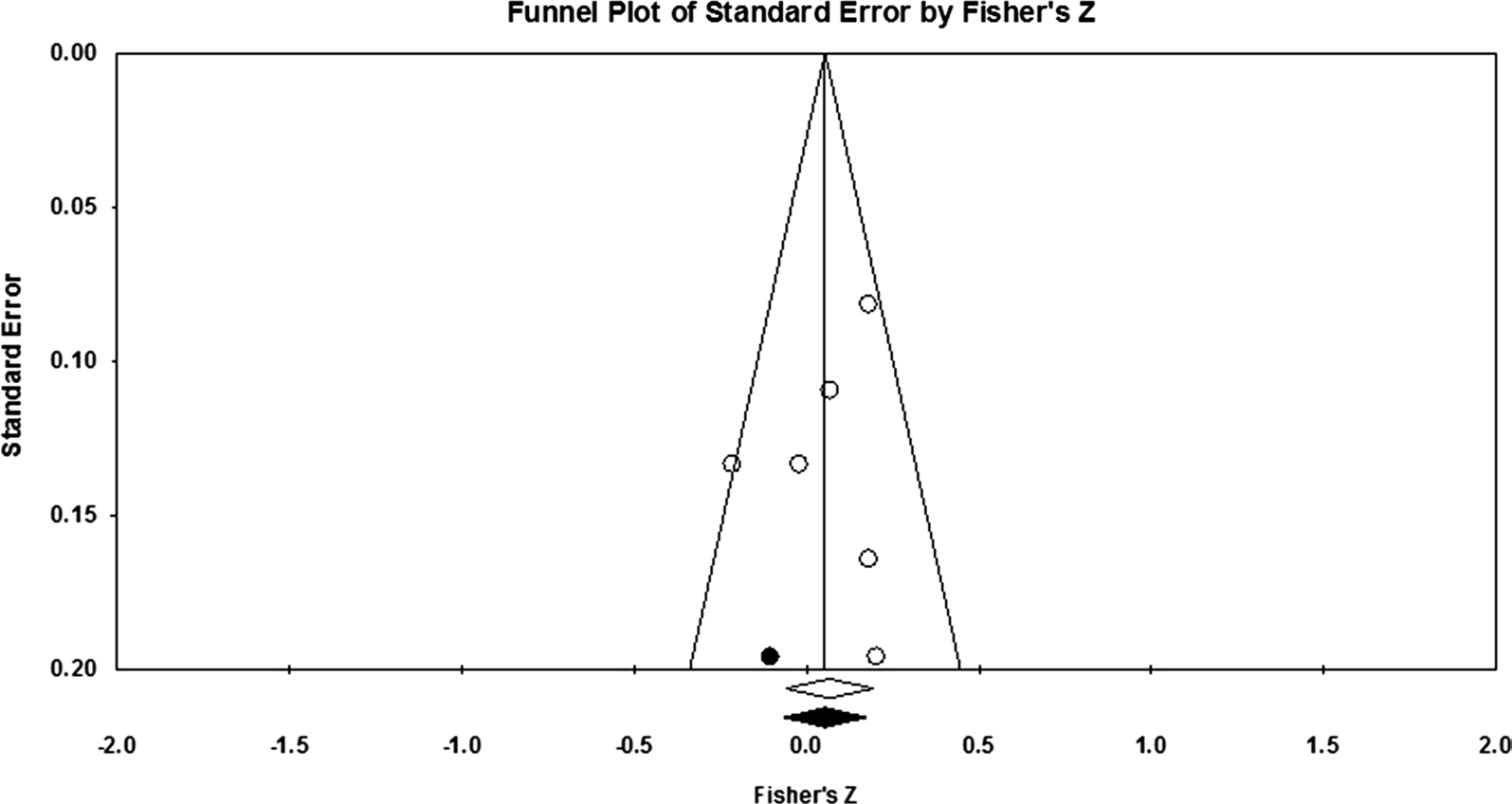
Funnel plot: Publication Bias and Average IQ. Note. The funnel plot demonstrates standard error estimates from actual and imputed (e.g., missing) studies; the former is indicated by white and the latter is indicated by black dots. The black-filled dot to the left of the axis suggests a study that shows a negative correlation between average member IQ and the criterion task could be missing from this review (e.g., file-drawered)

The distribution of standard error for the Fisher’s *Z* scores (actual is white, and imputed is black) in the funnel plot (Fig. 6) is consistent with the underreporting of at least one study showing a negative correlation between average IQ and the criterion task. Despite the low number of independent samples (*K* = 6) making this test somewhat unreliable, the mean estimate and range of the funnel fell well below the critical values (*Z* for alpha = 1.96 and .10 for a “trivial” correlation). Both the Classic fail-safe *N* and Orwin’s fail-safe *N* tests, which benchmark against these thresholds, indicate no additional studies are required to nullify the point estimate (mean) *Z*-value (1.33). This suggests the number of studies missing from this analysis is likely to be zero.

The notion of statistical power is now considered and relies on the conventions and guidelines first outlined by Cohen (1977,1988) and later expanded by Rosenthal and Rosnow (2008). Assuming an α (type-1 error probability, two-tailed) set at .05 and power (1—*β*) set at 80% (.80, 2-tailed) the number of groups necessary for detecting a small to moderate association (*r* =  .10 to .30) is calculated using the statistical G*Power 3.1 and ranged from 782 to 84 groups, respectively. Moreover, only 6 out of 19 (31.6%) samples fell within this range, while no samples exceeded it (0% > 784 groups). Under the same assumptions, the sample size necessary to detect a large effect (*r* ≥  .50) was estimated to involve at least 29 groups, and all but two of the 19 samples exceeded this level of statistical power. Indeed, the average power across all 19 samples for detecting a small (*r* ≥  .10 <  .30), moderate (*r* ≥ .30 < .50), or large (*r* ≥ .50) association between various operationalizations of intelligence (e.g., the *c-*factor or average IQ) and the criterion tasks was 13.3%, 61.8%, and 92.7%, respectively (see Table 5).

**Table 5:** The relationship between statistical power and observed and expected effect sizes

Power (%) by Effect Size						
Study	Sample size (groups)	Small (*r* ≥ .10 < .30)	Moderate (*r* ≥ .30 < .50)	Large (*r* ≥ .50)	c-factor (*r* = .26) ^a^	Mean IQ^b^ (*r* = .28)
Woolley et al. (2010 ): Study 1	40	9.4	47.7	92.0	37	41.9
Woolley et al. (2010 ): Study 2	152	23.3	96.6	100.0	90.1	93.8
Engel et al. ( [Bibr CR35][Bibr CR126]): Face-to-face (speaking) condition	32	8.4	39.2	85.3	30.3	34.3
Engel et al. ( [Bibr CR35][Bibr CR126]): Online (text-chat) condition	36	8.9	43.6	89.3	33.7	38.14
Engel et al. (2015a, b): Study 2 (Germany)	116	18.8	91.1	100.0	80.8	86.2
Engel et al. (2015a, b): Study 3 (Japan)	25	7.6	31.3	74.9	24.2	27.4
Woolley and Aggarwal (under review); (Also reported in Woolley and Aggarwal 2017)	59	11.7	64.6	98.5	51.5	57.7
Meslec, et al. (2016)	30	8.2	37.0	82.8	28.6	32.3
Glikson, Harush, et al. (under review)	115	18.6	90.8	100.0	80.4	85.9
Chikersal, et al. (2017)	58	11.6	63.9	98.4	50.8	56.9
Kim et al. ( [Bibr CR60][Bibr CR128])	248	35.0	99.8	100.0	98.6	99.4
Aggarwal et al. (2019)	98	16.5	85.8	100.0	73.8	79.9
Barlow and Dennis (2016 )	86	15.0	81.0	99.9	68	74.4
Barlow (2015, unpublished doctoral thesis): Control Group (CG)	64	12.3	68.3	99.0	54.9	61.3
Barlow (2015, unpublished doctoral thesis): Experimental Group (EG)	65	12.4	69.0	99.2	55.6	62
Bates and Gupta (2017): Study 1	26	7.7	32.5	76.7	25.1	28.4
Bates and Gupta (2017): Study 2	40	9.4	47.7	92.3	37	41.9
Bates and Gupta (2017): Study 3	40	9.4	47.7	92.3	37	41.9
Rowe (unpublished doctoral thesis)	29	8.1	35.9	81.4	27.7	6.4
Proportion of studies with acceptable (≥ 80%) power:		0 of 19 (0%)	6 of 19 (31.2%)	17 of 19 (89%)	4 of 19 (21.1%)	4 of 19 (21.1%)

*Note*: Calculations of statistical power are based on the actual sample size for included studies. All power calculations are written as percentage terms (%); Categories for the magnitude of association are based on the conventions of Cohen (1988); calculations are made using G*Power 3.1 software; correlations are for bivariate normal models (Pearson's *r*) and computed post hoc based on alpha error probability of .05 (two-tailed) and power of 80% (1—*β* = .80); power calculated based on tests against a null model (*r* ~ 0). Sample size is based on the actual number of groups included in the study and/or condition

^a^This value is based on the sample weighted correlation derived from the meta-analysis reported in Fig. 3

^b^This value is based on a sample weighted correlation derived from three meta-analyses investigating the relationship between average (or sum) IQ scores and group performance (Bell 2007; Devine and Philips 2001; Stewart 2006)

Two additional correlations were considered for post hoc power analysis. The first is based on the results of the present review which found a sample weighted correlation of .26 for the correlation between the *c-*factor and the group criterion tasks. The second correlation is based on a synthesis of three meta-analyses measuring the relationship between individual IQ and various group performance outcomes (Bell 2007; Devine and Philips 2001; Stewart 2006), which generated a sample weighted correlation of .28. Therefore, the statistical power necessary to detect an equivalent association was estimated for each sample (Table 5). Only 21.1% of studies were found to have the statistical power necessary (1–*β* ≥ .80) to detect an association, if one exists in the population, with the *c-*factor or average IQ. Put another way, around 80% of studies included in this review did not have enough statistical power to reliably detect associations between the primary predictor variables and the criterion outcomes.

## Discussion

This review attempted to synthesize quantitative results pertaining to the validity of the *c-*factor (or lack thereof), its relationship to the *g-*factor, and its effect, if any, on group performance tasks external and subsequent to the group IQ test battery. Ideally, the validity of a psychological construct should be established within the context of a nomological network of converging evidence relative to competing constructs (Cronbach and Meehl 1955). For example, the theory of general intelligence in individuals has little falsifying evidence while having amassed converging evidence verifying its existence from a quantitative, objective, analytical, biological, and socio-cultural perspective (Gottfredson 2016; Jensen 1986). These theoretical foundations provided the guideposts for exploring the validity of the *c-*factor across five different dimensions of effect in the present review. This acknowledges the many strands of evidence necessary to build a nomological network of construct validity. By the same token it takes but a few “broken strands” for a theory to come unstuck which, in a general sense, amounts to the most probabilistically determinate evidence science has to offer (Popper 2002). The results from this quantitative synthesis, though somewhat mixed and reflective of a topic in its earliest stages, do not necessarily bode well for the validity of the *c-*factor.

These sentiments were shared by one reviewer who advised that it is not “particularly informative to conduct a meta-analysis of research based on this fundamentally flawed idea” (i.e., the *c-*factor). However, the primary purpose of the present inquiry was to explore *“what evidence supports the validity of the c-factor”* (neither to assume it is valid nor fundamentally flawed) and compare it to its individual analogue, the *g-*factor, from which the *c-*factor found its conceptual, theoretical, and methodological inspiration (see research questions 1 and 2). In our opinion, the case against the *c-*factor, or against the possibility of an alternative general factor in groups (*c*, *g*, or otherwise), has *not* been firmly established. This is especially evident in relation to the predictive validity of the *c*-factor where it appears to show some promise in its practical application despite having obvious theoretical insufficiencies.

Nevertheless, at least one thorough criticism of the *c-*factor, by Credé and Howardson ([Bibr CR22][Bibr CR124]), raises serious doubts about its validity. They argued that researcher degrees of freedom in the analytic process may grossly distort the capacity to draw valid inferences from the data. Using simulations, they demonstrated that it was possible to partition the variance derived from individually nested data from the groups’ individual members and, in so doing, obliterate the lion’s share of common variance at the group level, leaving only task-specific variance unaccounted for. Credé and Howardson’s ([Bibr CR22][Bibr CR124]) arguments, however, hinged heavily on irregularities in the correlation matrices; specifically, that the matrices occasionally showed low and negative coefficients among the six studies included in their review. But uniformly high intercorrelations among group IQ items alone are insufficient in determining factor structure. These statistics can be easily inflated (e.g., see the notion of the “bloated specific” by Cattell 1978) and consequently require interpretation through a strong theoretical, methodological, and practical lens.

Factor-driven models often succumb to “an infinity of mathematically equivalent solutions” that lay at the mercy of researchers (Kline 1993, p. 11). The field of intelligence research, for instance, is replete with diverse factor models (e.g., Cattell 1963; Johnson and Bouchard 2005; McGrew 2009; Spearman 1927) and has been accused of being founded upon little more than a statistical artifact on more than one occasion (e.g., Horn and McArdle 2007; Kovacs and Conway 2019). Indeed, any number of “mathematical abstractions” that “bear no necessary relationship to anything in the real world” can be made of sound statistical solutions. This indicates external evidence, and not just statistical rigor, is “necessary to identify a factor convincingly” (Kline 2013, pp. 26–27). Therefore, the otherwise intractable problem of mathematically equivalent accounts of the data can be overcome by transitioning from explanatory to predictive models (Yarkoni and Westfall 2017). In the case of the *g-*factor, objections to its realness have typically crumbled under mounting evidence confirming *g*’s criterion validity beyond contrived testing or statically abstracted settings (Jensen 1998). It is in this spirit that we found previous reviews lacking, and thus directed our attention toward the relative efficacy of *g* and *c,* and their respective composites (i.e., group IQ scores), to predict meaningful variance in group performance outside of the group IQ testing environment.

The predictive validity of the *c-*factor in the present review was demonstrated by a small to moderate correlation with group performance across a variety of criterion-relevant tasks. Of the nine independent samples included in the meta-analysis pertaining to the correlation between the *c-*factor and criterion performance, five showed entirely positive 95% CIs (above zero), while three had 95% CIs partially or fully distributed below zero (see Fig. 3). Heterogeneity was considerable across studies investigating the effect of the *c-*factor on criterion tasks [*Q*(8) = 59.36, *p* < .001, *I*^2^ = 86.5%], suggesting sample effect estimates may not necessarily reflect the same latent scale and, therefore, not be readily interpretable using meta-analytic methods until further studies are included. The *c-*factor also showed incremental predictive validity over the *g-*factor, where results demonstrated a near-zero correlation between the average IQ of the group and a variety of criterion tasks from overlapping studies and samples (Fig. 5). The dispersion of results across studies was moderate for the correlations between average IQ and criterion tasks [*Q*(5) = 7.72, *p* = .172, *I*^2^ = 35.23%], suggesting heterogeneity did not render estimates unsuitable for meta-analysis for this effect.

The apparent superiority of the *c-*factor over the *g-*factor in predicting group performance does not necessarily correspond with several meta-analyses, including studies in both laboratory and field settings, which found the average IQ of groups shares correlations between .20 and .40 with a variety of criterion-relevant outcomes (Bell 2007; Devine and Philips 2001; Stewart 2006). The most surprising finding, however, was not that individual IQ shared almost no relationship with the external criterion tasks, but that it shared little to no relationship with many of the group IQ tests which were, under the present research paradigm, supposedly tapping a highly similar latent construct at both individual and group-levels (i.e., general intelligence). Yet the *c-*factor and average IQ scores shared only weak to moderate correlations, ranging from −.05 to .34 (see effect 4, Table 1). Indeed, factor loadings may reflect *fairly similar* latent constructs when correlations range from .85 to .94, and the *same* latent construct when correlations are ≥ .95 (Lorenzo-Seva and ten Berge 2006, p. 62). The correlations between the *c-*factor and *g-*factor fall far short of these thresholds and, if taken at face value, substantiate the case that they are empirically distinct constructs. A more nuanced interpretation of these findings that considers various methodological shortcomings may suggest otherwise. For example, the context in which Yarkoni and Westfall (2017) advocate for psychological researchers to embrace predictive over explanatory science is also one that prizes real-world results over those derived from laboratory settings. While effects 3 and 5 considered the *c* and *g*-factors, respectively, in relation to their predictive validity, many of these effects originated in laboratory contexts using contrived tasks and therefore may have lacked currency when applied to real-world settings where group-based activities hold much of their value (see Table 4).

### Problems with the group IQ paradigm

There are several reasons to believe that many of the studies in this review were unfit for testing the validity of the *c-*factor in groups. Firstly, different IQ tests were used across the individual and group conditions. This makes it entirely unclear how much of the total variance in group IQ tests that the *g-*factor accounted for at the group level. Moreover, the traditional (individual) IQ testing paradigm tends to require items to be psychometrically validated around various domains of mental ability, such as visual processing, reading and writing, and fluid reasoning (e.g., McGrew 2009). Additionally, a good IQ test will be mentally rather than physically exhaustive, offer a balanced range of mental tasks that adequately cover different dimensions of content, complexity, and mental operations, and be objectively verifiable rather than judgmental or probabilistic in nature (Gordon 1997; Jensen 1998).

Many of the group IQ tests included in this review violated these criteria in critical ways. Barlow and Dennis (2016), for example, only used three tests, and each was arguably lacking in any number of the above criteria. This is unsurprising given that the group IQ testing paradigms embraced in the present review almost exclusively sampled items based on their subjective alignment with various quadrants of McGrath’s (1984) task circumplex. McGrath’s circumplex is a group task taxonomy based on four qualitatively distinct task types: generate (quadrant 1), choose (quadrant 2), negotiate (quadrant 3), and execute (quadrant 4). Researchers who wish to sample tasks according to this circumplex presumably do so primarily based on conceptual and aesthetic grounds, which lack empirical standards by which items can be objectively distinguished. Results from a meta-analysis by Bell (2007) suggested that even the most popular group task typologies, such as Steiner’s (1972), which classifies group tasks according to the different ways individuals combine contributions toward group-related outcomes, have little to no moderating effect on the relationship between intelligence and group performance. This suggests that when researchers, such as Woolley et al. (2010), claim to have sampled items from “a wide variety of cognitive tasks” (p.686), but do so exclusively on the basis of typologies that are demonstrably problematic and originate outside of intelligence testing paradigms, they may in fact be doing an insufficient job covering the hypothesized construct, particularly in terms of the latent properties driving the cognitive operations behind group tasks.

Secondly, the design features required to employ multilevel factor analysis to address the nested nature of individual intelligence as it relates to collective intelligence were often violated. For example, a minimum of nine evenly balanced items are required to generate three first-order factors, from which a second-order general factor can be extracted (Jensen 1998). Yet only 2 of 19 studies met this criteria and neither reported details on the validity, reliability, or criterion relevance of the items in terms of their suitability for use in a group IQ test. This suggests many analyses may be tained by these items as they would be unlikely to reveal a “true” general factor if it were to emerge under either individual or group conditions. Finally, not only were the studies chronically underpowered (increasing the chances of type-I/II error, see Table 5), but also lacked the degrees of freedom required to reliably employ multilevel modeling. The importance of this approach cannot be overstated and is discussed at length in the review by Credé and Howardson ([Bibr CR22][Bibr CR124]). Consequently, the extent to which individual effects (i.e., *g*) are nested within group-level effects (i.e., *c*) is not easily examined under the constraints of the present dataset.

### Common factors with nothing in common

Despite the near-universal manifestation of a positive manifold among cognitive test items and the frequent extraction of a relatively large factor (or component) from them, the *c-*factor is not the only explanation for these findings. Subsequently, the results herein and the criteria used by Woolley et al. (2010) to establish the validity of the hypothesized *c-*factor are compatible with any number of alternative common-factor solutions. Others have previously hinted at the existence of a general factor of group performance, though not necessarily one pertaining to the group’s intelligence, at least as far back as the 1970s. Hackman and Morris (1975), using data from a sample of 108 experimental groups, examined the relationship between 16 interaction-process categories (e.g., propose solution, clarify, seek evaluation) and three intellective task performance categories (group production, discussion, and problem-solving tasks). Interaction-process scores shared a canonical weight of .68 with combined performance scores across all three categories (ranging from .59 to .66), leading them to conclude that “substantial variation in group performance on intellective tasks is controlled by the nature of the group interaction process” (Hackman and Morris 1975, p. 10).

Another noteworthy rival to the *c-*factor was elucidated by LePine et al. (2008) in a meta-analysis comprising 138 studies exploring the concept of “teamwork process.” Sitting atop LePine et al.’s (2008) multilevel model was a single factor referred to as the Teamwork Process Factor (TPF), which was subsequently shown to correlate with team performance (uncorrected *r* = .27, as measured by supervisor and/or member rated team performance, quantity and quality of team output, and innovation), member satisfaction (*r* = .38), cohesion (*r* = .45 to .52), and potency (*r* = .56 to .63). LePine et al. (2008) concluded that the TPF is a higher-order construct that reflects “the overall quality of teamwork processes” and has significant implications for various dimensions of team performance (see p. 287).

Perhaps the most plausible candidate for a general factor of ability in groups, however, is an aggregated form of general intelligence possessed by the groups’ individual members. We refer to this as the “Group *g*” or *G*_*g*_. There are many ways of conceptualizing and operationalizing the *g-*factor as it manifests in groups—the *G*_*g*_. A study by Kosinski et al. (2012), for example, had individual participants complete an online version of Raven’s Progressive Matrices (RPM) IQ test. They then used the statistical mode to select answers derived from randomly generated pseudo-groups that were systematically varied from 2 to 20 members in size. Kosinski et al. (2012) found performance improved monotonically with group size, suggesting additional member inputs probabilistically favor the solution and attenuate error. In this study, individual participants achieved an average IQ of 122 (> 91st percentile) and groups of 12 averaged IQs equivalent to 145 (> 99.6th percentile) (Kosinski et al. 2012). This is partly explained by the fact that the RPM tasks, which involve eight multiple-choice options, share a uniform prior binomial probability distribution of correctly solving the problem at a rate of 12.5% per event. As long as the probability of one or more members knowing what *is* and *is not* correct exceeds the random distribution, then additional members should improve the likelihood that a “majority vote” or “mode” decision model will succeed (see Laughlin 2011). It is against these outcomes, where solutions are generated without social interactions, that some have suggested the true utility of groups, including process losses and gains, can be gauged (e.g., Sears and Reagin 2013). This is because results that exceed this benchmark provide a clear answer to the question of when groups are likely to be more efficacious than individuals in discrete problem settings. Indeed, the way individual IQ scores were aggregated provided a similar benchmark against which Woolley et al. (2010) argued for the superiority of the *c* over the *g*-factor in predicting group performance.

In the context of the present inquiry, estimates of *G*_*g*_ were inferred based on the average IQ scores observed in the groups’ individual members (i.e., a derived variable). Despite being widely employed by researchers interested in group performance (e.g., Barrick et al. 1998; LePine 2005), averaging IQ scores is problematic because it potentially obscures information about the true *G*_*g*_—which may not necessarily map neatly onto the same latent scale as its namesake at the individual level, *g*. For example, averaging individual IQ overshadows within-group/between-individual variability, effectively nullifying the differential effect of *g* among team members and thus distorting estimates of true *G*_*g*_. A group with an average IQ of ~ 115 may exhibit low (e.g., IQ scores of 115, 116, and 114) or high (e.g., IQ scores of 130, 115, and 100) *within-group variability* (in this example, there is a 15-fold increase in the standard deviation of the latter compared to the former group). Almost all studies included in the present inquiry overlooked these differences and instead relied on between-group variability estimates, which circumvent potentially valuable information about the consistency of the groups’ performance dispositions.

Reinforcing these concerns are findings relating to the differential effects of cognitive ability in team performance contexts. For example, gains in group performance tend to disproportionately benefit lower ability members compared to higher ability members, and this pattern is likely to be moderated by time and the number of performance trials (Day et al. 2005; Goldman 1971; Lasek 1994; Laughlin and Branch 1972; Laughlin and Johnson 1966). Similarly, studies examining the effects of different combinations of ability grouping in educational settings (e.g., high, high, and low) have demonstrated that within-group heterogeneity powerfully moderates academic outcomes for students exposed to this pedagogical intervention (Lou et al. 1996). Some evidence also suggests that group performance measures are more sensitive to *between-group variability* at the lower end of the cognitive ability distribution such that groups with lower levels of average IQ tend to show stronger correlations with group performance than do groups with higher levels of average IQ (Bell 2007). A similar phenomenon has been observed with individuals, where more predictive value is contained in lower compared to the upper end of IQ distributions (e.g., Hegelund et al. 2018). The complement of this is known as the *cognitive ability differentiation hypothesis* (also known as Spearman’s Law of Diminishing Returns) which predicts correlations between cognitive ability tests to be weaker and less *g* loaded for those who perform at the upper end of the IQ distribution (Blum and Holling 2017). It is plausible that samples included in this review were above average in intelligence, which could have simultaneously suppressed criterion validities (i.e., via ceiling effects) and/or inflated unique variance in the correlation matrices (i.e., via ability differentiation effects).

This raises the possibility that averaging members’ *g-*loadings, rather than their IQs, may provide a more precise estimate of *G*_*g,*_ because factor loadings in the latter instance include common and unique variance elements of individual intelligence in the correlational estimates. An additional advantage of using aggregated *g-*loadings is that it may come closer to an “apples with apples” comparison between *g* and *c*. While several studies included in this review intended to compare individual and collective intelligence, they did so with an “apples and oranges” comparison by correlating average IQ or *c-*factor scores with criterion tasks (Fig. 3 vs. 5). This would have almost certainly disproportionately penalized the coefficients pertaining to the *g-*factor (i.e., average IQ scores) and its relative effect on group performance compared to the *c-*factor.

This makes the series of studies by Bates and Gupta (2017) especially striking because, despite inheriting many of the methodological shortcomings of the studies they intended to replicate (e.g., Woolley et al. 2010), the average IQ of the groups’ individual members was found to account for over 80% of the total variance in the group IQ test scores. The authors concluded that, rather than having little to no relationship, as was the case in the studies by Woolley et al. (2010), individual and group IQ scores covaried in a way that was “indistinguishable from 100%” (p.52). These findings not only undermine the validity of the *c-*factor, but also challenge long-held views among organizational researchers about the situational specificity of group performance (e.g., Cohen and Bailey 1997; Devine 2002; Hollenbeck et al. 2012). According to this view, group performance is determined by the interplay of unique combinations of compositional (e.g., skills, abilities), emergent (e.g., collective efficacy), process-related (e.g., communication rituals), and contextual (e.g., task types, reward systems) factors (Mathieu et al. 2008).

Yet the *g-*factor, which is thought to be situationally robust (e.g., Schmidt and Hunter 1981,1998) and highly stable across the human lifespan (e.g., Deary et al. 2013), should, in principle, retain certain aspects of these characteristics at the group level of analysis such that the *g-*factor, even when averaged across group members, shares a significant relationship with true *G*_*g*_ (see Kozlowski and Klein 2000, for arguments in support of this view). In addition to Bates and Gupta (2017), another study that directly tested and supported this hypothesis was conducted by Imbimbo et al. (2020) who asked 550 high-school students to complete alternative (odd/even) sets of Raven’s Advanced Progressive Matrices, first as individuals, then as 110 randomly allocated groups comprised of five members each. A generalized linear mixed model revealed a strong relationship between the probability function of selecting the correct answer at the individual level and selecting the correct answer at the group level using a majority vote method. Nevertheless, one ought to be cautious to avoid the *atomistic fallacy* by flippantly making inferences at the group level based on data collected at the individual level (Diez Roux 2002). Researchers such as Bates and Gupta (2017), along with a host of others (e.g., Barrick et al. 1998; Bell 2007; Devine and Philips 2001; LePine 2003,2005; LePine et al. 1997; Stewart 2006), offer findings that affirm the central importance of the *g-*factor in groups more broadly, but they lack consensus about its precise nature and expression in group performance contexts and thus raise important questions for future research in this area. Consequently, how true *G*_*g*_ manifests in group settings is not just a statistical exercise but an empirical proposition that should be systematically tested under a multitude of moderating and mediating conditions.

## Limitations

The present inquiry was limited in a variety of ways. Small samples effects and insufficient statistical power may have muddied the present analysis and inadvertently created differential reliabilities and spurious outcomes (Wiernik and Dahlke 2020). For example, only four studies comprising six independent samples (*n* = 366) adequately controlled for individual intelligence when investigating the effect of the *c-*factor on external criterion tasks (Rowe 2019; Woolley and Aggarwal, under review; Woolley et al. 2010). Furthermore, according to Cohen’s 80% power convention, only 21% of studies had enough statistical power to reliably detect the observed effect. This figure reflects a much broader issue underlying the replicability of research in psychology where, based on a large sample of 200 meta-analyses involving over 8000 papers, it was estimated that only 8% of studies included in meta-analyses have sufficient power to reliably detect the targeted effect; most results are highly variable (the median *I*^2^ was 74%), and median levels of observed power are low at 36% (Stanley et al. 2018). Though meta-analyses have often been sought to overcome issues associated with small samples, Stanley et al. (2018) argue that the probability of type-I/II error may aggregate rather than attenuate in meta-analyses, particularly when high levels of heterogeneity are incorporated into these models.

It is also possible differences in the quality of the studies may have affected the manifest relationship between *g* and *c* and the meta-analytic syntheses herein. Ceiling effects in individual IQ scores, for example, may have partly concealed the effect of individual IQ in at least one study (see Woolley and Aggarwal, under review). Likewise, the failure to control for differential individual participation rates during the group activities makes it possible that the *c-*factor merely reflects the mental efforts of only one or a small proportion of individuals rather than the whole group per se. This may unduly penalize the comparative validity of the *g-*factor because its operationalized counterpart, average IQ, is weighted equally across all group members even when group participation is not. In contrast, the *c-*factor is derived from and thus attenuated by results from the group IQ tasks and, coupled with non- or low-participating members, may lead to inflated correlations between the *c-*factor and the group IQ composite. Considering this, it may also be reasonable to expect non- or low-participatory patterns to persist from the group IQ testing conditions into the criterion tasks, further inflating the relative correlations of the *c* compared to the *g-*factor.

Finally, all included studies *conceptually* rather than *directly* replicated the original studies by Woolley et al. (2010). Though conceptual replications often provide the impression of broadening established findings, they may also, particularly if conducted apart from a solid foundation of direct replication research, cause a field to become “grossly misled about the reality of phenomena” and inflate one’s sense of the general replicability and generalizability of a psychological construct (Pashler and Harris 2012, p. 533). Yet direct replication of studies such as Woolley et al. (2010) may be prohibitively expensive and thus “remain castles in the air, leaving us with little insight about replicability rates” (Laws 2016, p. 3).

## Conclusion

The notion of group performance does and should fascinate all of us; groups are an exciting yet poorly understood vehicle of change in the modern work and educational context. However, we should be cautious, despite its enormous appeal and potential, of embracing the *c-*factor as a panacea for better understanding and improving group performance. Claims that the *c-*factor, “has been well established in the literature” (Askay et al. 2019, p. 492) are incommensurate with the present body of evidence, which uncovered more questions than answers. Though a superficial view of the evidence herein may suggest the *c-*factor is a valid predictor of group performance, while the *g-*factor is not, a host of methodological shortcomings belie this point and do not bode well for the overall validity of the *c-*factor.

Chronically low-powered studies, major issues with the design features of the group IQ test batteries (e.g., psychometrically irrelevant, unreliable, and an insufficient number of items), a paucity of direct and/or independent replications, a failure to account for non-participation rates, and inadequate controls for the *g-*factor all served to undermine arguments both for and against the *c-*factor. Of course, one may correctly argue that it is the role of a scientist to remain skeptical and assume the validity of the null hypothesis unless evidence points to an alternative. But in this case, there were stark differences in how similar datasets were interpreted among competing models rather than a null model per se. Indeed, what is considered by one researcher as confirmatory can be interpreted by another researcher as disconfirmatory evidence (e.g., Botvinik-Nezer et al. 2020; Silberzahn et al. 2018).

Credé and Howardson ([Bibr CR22][Bibr CR124]), for example, share the prevailing view among organizational researchers (e.g., Devine 2002) that suggests group performance is a situationally specific phenomenon. This not only eliminates the possibility of a *c-*factor but is incompatible with alternative general factors of group performance such as LePine et al.’s (2008) team process factor, Hackman and Morris’ (1975) interaction-process ability and, most relevant to the present inquiry, a socially aggregated form of general intelligence, *G*_*g*_, that emerges from the groups’ individual members.

It is the latter alternative, the *g-*factor of the groups’ individual members, that was advocated by Bates and Gupta (2017) and provides the most credible challenge to the situational specificity hypothesis in group performance settings. The methods of operationalizing the *g-*factor in groups to enable an accurate estimation of true *G*_*g*_ is a highly challenging endeavor. While many have settled for merely averaging the IQ scores of the groups’ individual members, this approach almost certainly fails to capture vital information about how the *g-*factor truly manifests in group performance settings and the latent scale upon which it operates (i.e., true *G*_*g*_). Assumptions that *g* can be partitioned out of multilevel models based on individually derived parameter and variability estimates may also be highly problematic, particularly if its properties at the individual level do not hold at the group level (per the atomistic fallacy). As one reviewer mentioned, many of the issues surrounding the “explanatory success” and/or “reality” of various constructs may be adjudicated by using predictive accuracy as a benchmark for validity. Future research into the validity of the *c*-factor (and *g*-factor) in group performance settings would do well to integrate such criteria into the initial study design, with predictions about various real-world and out-of-sample group performance tasks taking precedent over those which are both experimentally contrived and operated within the confines of a laboratory (see Yarkoni and Westfall 2017). Moreover, innovative approaches to measuring group ability, such as Cookes’ method, should be considered. This approach advocates for group performances to be weighted according to how the individual members of the group perform during a calibration phase that, for example, may involve measuring one’s IQ to serve as a baseline weight, although there may not be enough evidence to gauge the utility of innovative compared to traditional methods (Steyvers and Miller 2015).

Looking to the future, rather than arguing for a conceptually and empirically distinct *c-*factor at the group level, researchers may be better served by systematically exploring more reliable and valid methods of operationalizing and analyzing the *g-*factor as it manifests in groups, particularly in relation to its many moderators in social settings (e.g., group size, group type, problem type, ability grouping, participation rates, interaction patterns). Until this is achieved it may be impossible to disentangle the *g* from the *c-*factor. In the meantime, we should remain skeptical about the *c-*factor and continue to recognize what has been obvious for decades: smarter groups tend to be those comprised of smarter individuals (Bates and Gupta 2017).

## Data Availability

Full references for the included list of studies, datasets analyzed, and RStudio scripts used to analyze the current study are available in the Open Science repository, https://osf.io/xevkj/

## Abbreviations

CI: Collective intelligence
*c-*factor: A general factor of collective intelligence.
*g-*factor: A general factor of individual intelligence
*G*_*g*_: A group’s general intelligence, *g*, based on that of its individual members
